# Pharmacological and non-pharmacological treatments for refractory paediatric Still’s disease: a scoping review

**DOI:** 10.1093/rap/rkaf123

**Published:** 2025-11-18

**Authors:** Elizabeth Twynam-Perkins, Neil Martin, Kirsty McLellan

**Affiliations:** Department of Paediatric Rheumatology, Royal Hospital for Children, Glasgow, UK; Department of Paediatric Rheumatology, Royal Hospital for Children, Glasgow, UK; Department of Paediatric Rheumatology, Royal Hospital for Children, Glasgow, UK

**Keywords:** systemic juvenile idiopathic arthritis, Still’s disease, refractory, macrophage activation syndrome, interstitial lung disease

## Abstract

**Objectives:**

Management of systemic juvenile idiopathic arthritis, or Still’s disease, has been transformed by the introduction of IL-1 and IL-6 antagonists. Despite this, a minority have refractory disease with three subtypes described: (1) persistent arthritis, (2) recurrent or difficult-to-treat macrophage activation syndrome (MAS), or (3) interstitial lung disease. This scoping review aimed to summarize available evidence for the treatment of refractory paediatric Still’s.

**Methods:**

Databases were searched using terms ‘systemic JIA’ or ‘Still’s disease’ AND ‘refractory’, plus synonyms. Records were screened for papers on the treatment of refractory Still’s, before reviewing full texts. Controlled trials, uncontrolled trials and case series/reports involving patients with disease onset <16 years were included. Data were extracted in tabulated form for study characteristics and outcome measures (survival, disease remission, reduction in corticosteroids, and adverse effects). Quality assessment was performed using the appropriate JBI checklist.

**Results:**

Thirty articles matched the inclusion criteria: 1 pilot study, 8 case series and 21 case reports. No controlled studies were identified with all three subtypes of refractory Still’s represented. There were positive results in a pilot study of emapalumab (IFN-gamma inhibitor) for the treatment of refractory MAS, and beneficial results with combination biologics and Janus kinase (JAK) inhibitors for refractory Still’s arthritis. There are reports of successful management with allogenic stem cell transplantation, although with significant risks.

**Conclusions:**

Various treatment strategies are reported in refractory Still’s, including emapalumab, JAK inhibitors, rituximab and combination biologics, although the evidence base is currently limited. Consistent outcome reporting and innovative trial designs are required to provide further evidence in these challenging subgroups.

Key messages

- The use of a range of biologic and non-biologic therapies has been reported in patients with refractory paediatric Still’s disease, but the evidence base at present is limited, with a lack of controlled trials and inconsistent reporting of outcomes.
- There are indications of efficacy with JAK inhibitors, rituximab and combination biologics in refractory Still’s arthritis, and there have been promising results with emapalumab (an IFN-gamma inhibitor) in refractory MAS.
- Consistent outcome reporting and innovative trial designs are required to provide further evidence for the treatment of these challenging subgroups of patients with Still’s disease.

## Introduction

Systemic juvenile idiopathic arthritis (sJIA), or Still’s disease, is a complex disorder characterized by systemic inflammation, with features of fever, rash, lymphadenopathy, serositis and hepatosplenomegaly, and concurrent or subsequent arthritis [1]. It can be complicated by macrophage activation syndrome (MAS), a hyperferritinaemic syndrome associated with cytopenias, liver dysfunction, coagulopathy, central nervous system involvement and end-organ damage, and by interstitial lung disease (ILD); both complications have significant mortality [1]. 40% of patients have a monophasic course, but the remainder have either a polyphasic or chronic course [2].

The 2024 EULAR/PReS guideline outlined that sJIA and adult-onset Still’s disease (AOSD) should be considered part of the same disease spectrum, and reflecting this, we will herein refer to sJIA as paediatric Still’s disease [3].

Still’s disease is considered a polygenic condition, with both autoinflammatory and autoimmune involvement [4]. It has been demonstrated that innate immunity is key early in the disease, with increased monocyte activation, high levels of IL-1β, and increased IL-6 gene expression in the peripheral blood of affected patients [1, 5]. In genetically predisposed individuals, certain triggers activate inflammasomes through Toll-like receptors, leading to caspase activation, conversion of IL-1β and IL-18 to their active forms, and their release into the extracellular milieu through pore formation by gasdermin D [6]. In MAS specifically, activated macrophages and cytotoxic T cells interact, signalling via IL-18 and IFN-gamma [1]. Later in the disease, adaptive immunity becomes more prominent, with expansion of IL-17-producing CD4^+^ T cells in patients with chronic synovitis [7]. The ‘window of opportunity’ theory postulates that controlling the early autoinflammation may prevent adaptive immune activation, driving chronic, more difficult-to-treat autoimmune arthritis [8]. The pathogenesis of Still’s-associated ILD remains poorly understood. It is more likely in patients affected by MAS, demonstrating a similar cytokine profile of raised IL-18 and IFN-gamma, along with T-cell activation [9]. Increasing recognition coincided with the introduction of IL-1 and IL-6 inhibitors, raising concern that it represents a drug rash with eosinophilia and systemic symptoms (DRESS)-like reaction. An alternative explanation, however, is the ‘cytokine plasticity hypothesis’ whereby IL-1/IL-6 inhibition promotes Th1 differentiation of CD4^+^ T cells in susceptible individuals [9].

Historically, high-dose corticosteroids were the mainstay of treatment, but with a significant side effect burden [5]. Treatment approaches established in JIA have been less efficacious in Still’s disease, likely reflecting different pathophysiology. A historic randomized controlled trial (RCT) of MTX versus placebo showed no significant global improvement in the Still’s subgroup [10]. TNF inhibitors were shown to be less effective in systemic JIA, with benefit in under half of patients in one observational study [11, 12]. Ciclosporin continues to be used as an adjunct in Still’s, with historic observational evidence and more recent uncontrolled studies in resource-limited settings [13–16]. It appeared to be useful in controlling systemic features and in treating MAS, but less efficacious in managing arthritis [16–19].

Increased understanding of the pivotal role of innate immunity, with prominent IL-1 and IL-6 signalling, transformed the management of Still’s [5]. Most patients (60–80%) can achieve remission with early introduction of an IL-1 or IL-6 antagonist, with reduced corticosteroid burden [1]. This is supported by RCT evidence [20–23].

A subset of patients, however, have disease refractory to these treatments, perhaps up to 20% [24]. Several different refractory disease courses have been observed. Erkens et al. [2] defined three subtypes of difficult-to-treat Still’s:

1. refractory Still’s arthritis: persistent arthritis not responding to IL-1 or IL-6 inhibitors, requiring maintenance glucocorticoids to control arthritis;
2. refractory Still’s-related MAS: treatment-resistant MAS, requiring long-term glucocorticoid therapy, or recurrent MAS (>/=2 episodes)
3. Still’s-associated lung disease: ILD, pulmonary artery hypertension and/or pulmonary alveolar proteinosis, often in the context of MAS, presenting with digital clubbing, tachypnoea and diffuse abnormalities on chest imaging.

Similar definitions have been proposed by other groups, each having in common failure to respond to IL-1/IL-6 inhibition, glucocorticoid dependence, or difficult-to-treat complications [1, 24].

Presented here is a scoping review of management strategies for refractory paediatric Still’s. Conducting trials in a rare and life-threatening condition such as this is logistically and ethically challenging [25]. Inclusive selection criteria were used, with the aim of summarizing the available evidence.

## Methods

### Research question

The scoping review sought to answer the following question:

What is the evidence-based management of the three subgroups of refractory paediatric Still’s disease, as defined by Erkens et al.: (1) refractory Still’s arthritis, (2) Still’s-related MAS, (3) Still’s-related lung disease?

We aimed to include clinical trials, case series and case reports of patients with disease onset <16 years. The interventions reviewed included any pharmacological and non-pharmacological treatments. Data were collected on primary outcomes of survival and clinically inactive disease (CID)/remission, and secondary outcomes of reduction/cessation of corticosteroids and adverse effects.

### Search strategy

The OVID interface was used to search Medline and Embase using Boolean search terms. MeSH terms were used where available, whilst keyword searches were of title, abstract and keywords. The search was limited to papers in the English language. See Table 1 for search terms. The search was performed on 15 April 2024 with records exported to EndNote for screening.

**Table 1.** Search terms

Resource	Search terms
Medline	[Arthritis, Juvenile OR (juvenile idiopathic arthritis or still’s disease)] AND [systemic] AND [refractory OR ((difficult* or hard or fail* or no) adj3 (treat* or respon*))]
Embase	[(systemic juvenile idiopathic arthritis) OR (systemic juvenile idiopathic arthritis or still’s disease)] AND [refractory OR ((difficult* or hard or fail* or no) adj3 (treat* or respon*))] NOT [adult.ti]
Cochrane database	(systemic juvenile idiopathic arthritis AND refractory):ti, ab, kw

### Study selection

Duplicates were identified and removed by EndNote and the Systematic Review Accelerator Deduplicator software [22]. A single reviewer (E.T-P.) screened titles/abstracts before full texts were located and reviewed for alignment with the inclusion/exclusion criteria (Supplementary Table S1, available at *Rheumatology Advances in Practice* online) by authors E.T.P. and K.M., with cases of uncertainty (8.7%) discussed to reach a consensus.

### Data collection

Data were collected by a single reviewer (E.T-P.) for included studies using a pre-designed data collection proforma, on the following variables: intervention, previous treatments, concomitant medications, number of patients, age (range), duration of illness and outcomes as described above. Where described in original articles, the definition used for CID/remission is detailed in the table. Data were collected as reported, with study authors not contacted for further information. When case series included patients with various rheumatological conditions, the data for the patients with paediatric Still’s were extracted where possible.

### Assessment of bias

A risk of bias assessment was performed by the primary reviewer by applying the Joanna Briggs Institute (JBI) checklist for critical appraisal of case series to the reports, including three or more patients [23].

See Supplementary Data S1, available at *Rheumatology Advances in Practice* online for the review protocol.

## Results

### Results of search

A total of 762 records were found. After initial screening of titles/abstracts, 69 full texts were assessed for eligibility. Reasons for exclusion are detailed in the PRISMA flow chart (Supplementary Fig. S2, available at *Rheumatology Advances in Practice* online).

Thirty articles met the inclusion criteria. No controlled studies were identified. One single-arm pilot study and eight case series were found. The remaining papers were case reports.

### Characteristics of included studies

Eleven reports featured patients with the subtype of refractory Still’s arthritis, including four small case series. Twelve studies included patients with refractory Still’s-MAS, including the single-arm pilot study. Two case reports featured patients with Still’s lung disease, and two case reports featured a combination of recurrent MAS and lung disease. Three case series included a mix of subtypes.

Several case series included patients with other conditions, but tabulated data on individual patients allowed information on the patients with Still’s to be extracted. Patients ranged in age from 5 months to 30 years.

Interventions for refractory Still’s in the included studies were: ciclosporin, combination therapies, emapalumab (IFN-gamma inhibitor), etoposide, increased dosing of IL-1 antagonist, IL-18 blockade, Janus kinase (JAK) inhibitors, plasma exchange, rapamycin, rituximab and haematopoietic stem cell transplant (HSCT).

The outcome measures for clinical response/remission were heterogeneous between the reports, ranging from use of the Wallace criteria, to scales used in adult disease (DAS28; Modified Pouchot score), to statements on resolution of symptoms and laboratory parameters, on or off immunosuppressant medication.

### Risk of bias in included studies

Table 2 shows the risk of bias assessment using the JBI checklist [26] for the eight included case series with three or more patients with Still’s. Seven of the studies were assessed to be at moderate-to-high risk of selection bias due to the absence of/lack of clarity on the consecutive and complete inclusion of participants. Diagnostic criteria were stated to be used in only three of the studies. Outcomes were not clearly reported in two of the case series. Case reports are inherently at risk of reporting bias, with positive outcomes more likely to be reported. Moreover, many patients described in the reports were receiving concomitant medications, with potential for confounding.

**Table 2.** Quality appraisal of included case series, using JBI checklist

	De Benedetti	Horne	Jaime-Perez	Kostik—Canakinumab	Kostik—Tofacitinib	Narvaez	Record	Silva
Were there clear criteria for inclusion in the case series?	Y	Y	Y	Y	Y	Y	Y	Y
Was the condition measured in a standard, reliable way for all participants included in the case series?	Y	Y	Y	Y	Y	Y	U	Y
Were valid methods used for identification of the condition for all participants included in the case series?	Y	Y	U	Y	U	U	U	U
Did the case series have consecutive inclusion of participants?	Y	U	N	N	N	U	N	N
Did the case series have complete inclusion of participants?	Y	U	U	U	U	U	U	U
Was there clear reporting of the demographics of the participants in the study?	N	Y	Y	Y	Y	Y	Y	Y
Was there clear reporting of clinical information of the participants?	N	Y	Y	Y	Y	Y	N	Y
Were the outcomes or follow-up results of cases clearly reported?	Y	Y	Y	N	Y	Y	N	Y
Was there clear reporting of the presenting site(s)/clinic(s) demographic information?	N	N	Y	N	N	N	N	N
Was statistical analysis appropriate?	N/A	N/A	N/A	N/A	N/A	N/A	N/A	N/A

Y: yes, N: no, U: unclear, N/A: not applicable.

### Refractory still’s arthritis

Table 3 summarizes included papers describing treatments for refractory Still’s arthritis.

**Table 3.** Treatments for refractory Still’s arthritis

Refractory Still’s arthritis								
Study (author, year, location)	Type of study	Intervention	Previous treatment	Concomitant medications	Intervention (No. of patients; age)	Duration of illness (range); age at diagnosis (range)	Outcomes	Adverse effects
*Non-biologic medications*								
Concha, 2021, Chile [28]	Case report	Rapamycin 2 mg/m^2^ daily	i.v. methylprednisolone, oral corticosteroids, MTX, tocilizumab, infliximab, ciclosporin, canakinumab, tofacitinib	Oral corticosteroids, MTX	1 patient, aged 8 years	2 years; 8 years old at onset	CID by 3 months (arthritis remission, FBC, ferritin and inflammatory markers normalized)	None
							Oral corticosteroids reduced to physiological replacement dose	
*JAK inhibitors*								
**Kostik, 2022, Russia** [30**]**	**Case series**	**Tofacitinib 0.25–0.5 mg/kg**	**Corticosteroids, tocilizumab, canakinumab, anakinra in 1, abatacept in 1, etanercept in 2, tofacitinib in 1, ciclosporin in 1**	**Canakinumab in 2, tocilizumab in 1**	**4 patients, aged 4–15 years**	**Information not provided**	**All alive at 6 months**	**Not specified whether in Still’s patients or others: 4/24—liver enzymes elevation (*n* = 2), hypercholesterolemia (*n* = 1), lymphadenitis (*n* = 1)**
							**Complete response (Wallace criteria) in 1, partial response (at least 50% improvement in active joints, ESR, CRP, VAS for JIA) in 2, no response in 1**	
**Gillard, 2022, France** [31**]**	**Case series**	**Ruxolitinib 5 mg BD** **->15 mg** **BD** **Baricitinib** **4 mg/day** **->8 mg/day**	**CsDMARD, IL-1 inhibitor, IL-6 inhibitor, TNF inhibitor, other**	**Oral corticosteroids (5 mg/day and 40 mg/day)**	**2 patients with Still’s, aged 6 and 16 years**	**Duration 5–15 years; age at onset 1–2 years**	**Alive at 33 months and 12 months**	**Varicella zoster infection**
							**Complete remission (modified Pouchot score 0, no symptoms, normal CRP)**	
							**Corticosteroids reduced to 1 mg/day and 7.5 mg/day**	
*Biologics*								
**Narvaez, 2009, Spain** [32**]**	**Case series**	**Rituximab 1 g x2**	**Anakinra in all, anti-TNF in all; various non-biologic DMARDs**	**MTX in 2**	**3 patients, aged 29–30 (onset of Still’s in childhood)**	**Duration 18–27 years; age at onset 2–12 years**	**All alive at 1 year**	**Severe hypersensitivity reaction in 1**
							**Remission of systemic symptoms in all at 6 months** **Moderate response in DAS28 scores at 6 months**	
							**Corticosteroid dose reduced by more than half at 1 year**	
Kasher-Meron, 2009, Israel [33]	Case report	Rituximab 1 g x2	Anakinra, etanercept, infliximab, IVIG, MTX, cyclophosphamide, HCQ, ciclosporin, thalidomide	MTX then azathioprine	1 patient, age 18 years	Duration 12 years; age at onset 6 years	Sustained remission for 18 months (no systemic or active arthritic symptoms, CRP reduced to 1.5 mg/dl (slightly above normal))	None reported
							Weekly methylprednisolone dose reduced to 30 mg	
*Combination therapies*								
**Record, 2011, USA** [34**]**	**Case series**	**Abatacept + anakinra**	**Anakinra, corticosteroids,** MTX**; 1 also had ciclosporin, cyclophosphamide and IVIG**	**Prior medications initially continued**	**4 patients, aged 4–12 years**	**Duration 18 months–66 months; age at onset 8 months—10 years**	**All alive at 8 months**	**None reported**
							**Not consistently described. Partial response in all; arthritis resolved in 1, Joint count reduced by 70% in 1, no further MAS in 1, fevers reduced in 1.**	
							**Prednisolone dose reduced by 0.2–4 mg/kg/day.**	
Conti, 2023, Italy [35]	Case report	Tocilizumab + ixekinumab (anti-IL-17)	Corticoteroids, tocilizumab, canakinumab, anakinra, MTX, etanercept, ciclosporin, baricitinib	Prednisolone	1 patient, age unclear	Duration unclear (>1 year); age at onset 4 years	‘Control of the disease’	None reported
							Prednisolone tapered to 0.1 mg/kg/day at 18 months	
*Haematopoietic stem cell transplant and other cell-based therapies*								
**Silva, 2018, UK** [62**]**	**Multicentre case series/cohort study**	**Allogenic HSCT**	**Corticosteroids, MTX in 5, anti-TNFs in 4, rituximab in 2, ciclosporin in 1; all had anakinra/canakinumab and tocilizumab**	**Conditioning regime** **Ciclosporin** **MMF**	**6 patients, aged 4–16 years**	Duration 3–15 years; age at onset 9 months—12 years	**All alive at minimum 10 months’ follow-up**	**Viral infection/reactivation in 2, GVHD in 1, thyrotoxicosis in 1, immune thrombocytopaemia in 1**
							**Complete remission (symptom-free and off immunosuppressants) in 5/6, though one had a further flare at 22 months, resolving with a short course of steroids; partial remission (resolution of inflammatory symptoms, but ongoing immunosuppressants) in 1/6**	
Beaufils, 2021, 2023, Canada [55, 56]	Case report	Allogenic HSCT	Corticosteroids, MTX, tocilizumab, anakinra in 1, canakinumab, tofacitinib, infliximab in 1, ciclosporin in 1, siltuximab in 1	Conditioning Post-transplant immunosuppression (corticosteroids; cyclophosphamide or ciclosporin; MMF; ruxolitinib or tofacitinib; eculizumab or rituximab; adalilmumab in 1)	2 patients, aged 5 and 7 years	Duration 1.4–3.8 years; age at onset 3–4 years	Alive at 4 and 5 years’ follow-up	Inflammatory episodes, severe hypertension with PRES, GVHD, mucositis, renal failure, HSV reactivation, EBV reactivation
							Complete remission off immunosuppressant therapy in 5 years old; relapses controlled by multiple biologics in 7 years old	
Morelle, 2020, 2021 [60, 61]	Case report	Haploidentical allogenic HSCT	MTX, tocilizumab, anakinra, canakinumab, adalimumab, thalidomide, infliximab	Conditioning including rituximab, cyclophosphamide and ciclosporin	1 patient, aged 3 years	Duration 2 years; age at onset 15 months	Complete remission off immunosuppressive drugs 3 years	Bacterial sepsis, GVHD, C. Difficile, hepatitis, autoimmune thrombocytopaenia, thyroiditis, shingles
**Jaime-Perez, 2022, Mexico** [63**]**	**Case series**	**Autologous HSCT**	**Corticosteroids, MTX, tocilizumab, HCQ**	**Conditioning**	**1 patient with refractory Still’s arthritis, aged 6**	**Duration 2 years; age at onset 4 years**	**Alive at 11 months’ follow-up**	**Neutropaenic fever**
							**Complete remission (resolution of symptoms off immunosuppressive therapy), then relapse at 6 months requiring corticosteroids**	
Rodrigues Pereira Almeida, 2021, Brazil [64]	Case report	Autologous HSCT	Corticosteroids, MTX, leflunomide, etanercept, ciclosporin, adalimumab, tocilizumab, canakinumab, MMF, rituximab, cyclophosphamide, IVIG, abatacept	Conditioning including cyclophosphamide	1 patient, aged 15 years	Duration 9 years; age at onset 6 years	‘Sustained remission’	None reported
							Prednisolone tapered to low dose	
Swart, 2019, Netherlands [65]	Cohort study; 1 with Still’s	Mesenchymal stromal cells	Corticosteroids, MTX, ciclosporin, thalidomide, cyclophosphamide, tofacitinib, abatacept, anakinra, canakinumab, etanercept, infliximab, rituximab, tocilizumab	MTX, prednisolone, tocilizumab	1 patient, aged 16 years	Duration 12.7 years; age at onset (3 years)	Ongoing disease activity, including episode of MAS	None reported

Case series shown in bold. CID: clinically inactive disease; csDMARD: conventional synthetic disease-modifying anti-rheumatic drug; FBC: full blood count; GVHD: graft-versus-host-disease; HSCT: haematopoietic stem cell transplant; MAS: macrophage activation syndrome; PRES: posterior reversible encephalopathy syndrome; sJIA: systemic juvenile idiopathic arthritis; VAS: visual analogue scale.

#### Non-biologic medications

Conventional synthetic DMARDs (csDMARDs) such as MTX have a limited role in the management of systemic features of Still’s, though clinical experience shows that they can be beneficial in some patients for the management of arthritis [10, 27]. No papers reporting the use of csDMARDs in Still’s were found that met the definition of refractory disease.

One case report of a patient with refractory Still’s arthritis treated with rapamycin (also known as sirolimus) reported CID after 3 months [28]. To the best of our knowledge, use of rapamycin is not reported elsewhere.

#### JAK inhibitors

Tofacitinib has been approved for use in polyarticular JIA based on a withdrawal-design RCT [29]. A retrospective series of 24 paediatric patients treated with tofacitinib included 4 patients with refractory Still’s arthritis [30]. All were alive at a minimum of 6 months of follow-up. One achieved CID as per the Wallace criteria, two partial responses and one no response. Of note, two patients were continued on canakinumab (complete response, partial response) and one on tocilizumab (partial response). A small case series of adults and children with difficult-to-treat Still’s disease treated with various JAK inhibitors included two children with refractory Still’s arthritis, treated with ruxolitinib and baricitinib [31]. Both achieved complete remission after dose increases, with ongoing reduction of oral steroids at the end of follow-up.

#### Rituximab

A series of three adults with longstanding Still’s from childhood reported remission of systemic symptoms in all three, with moderate response in DAS28 [32].

One other case report met the inclusion criteria, with this 18-year-old patient achieving sustained remission for 18 months [33].

#### Combination therapies

One case series reported the combination of abatacept and anakinra in four children with refractory Still’s arthritis [34]. All survived, with improvements in clinical features, but outcomes were not consistently described.

A single case report described an HLA-B27-positive patient with difficult-to-treat Still’s arthritis treated with tocilizumab and ixekinumab (anti-IL-17), with improved disease control allowing tapering of corticosteroids [35].

### Refractory Still’s-MAS

Table 4 shows articles reporting treatments for refractory Still’s-MAS.

**Table 4.** Treatments for Still’s-associated MAS

Still’s-associated MAS								
Study (author, year)	Type of study	Intervention	Previous treatment	Concomitant medications	Intervention (number of patients; age)	Duration of illness (range)/age at diagnosis (range)	Outcomes	Adverse effects
*Non-biologic medications*								
**Horne, 2021, Sweden [** 24 **]**	**Case series**	**Etoposide, moderately dosed (50–100 mg/m^2^ once weekly)**	**i.v. methylprednisolone, oral corticosteroids, anakinra (3), ciclosporin (2)**	**Oral corticosteroids, ciclosporin (3), IVIG (2), anakinra (1), rituximab (1)**	**5 patients; 5 months–16 years**	**Duration 3–60 months**	**All alive at 2 years**	**Neutropaenic sepsis in 1/5, febrile neutropaenia in 1/5**
							**‘Complete response’ in all 1 developed recurrent MAS-> allogenic HSCT**	
							**All off corticosteroids at last follow-up (2–8 years after onset)**	
Khawaja, 2019, UAE [38]	Case report	Ciclosporin 100 mg twice daily	Anakinra, i.v. methylprednisolone for MAS; MTX, oral corticosteroids, etanercept, tocilizumab before MAS	Anakinra, i.v. methylprednisolone, IVIG	1 patient, age unclear	Duration unclear; age at onset 12 years	No benefit; starting cyclophosphamide at the time of report	None reported
*JAK inhibitors*								
Ocasio, 2021, Puerto Rico [39]	Case report	Tofacitinib	MTX, ciclosporin, tocilizumab, canakinumab	Unclear	1 patient, aged 5 years (timeline unclear)	Age at onset 18 months	Initial response, then recurrence of rash	None reported Later developed lung disease
*Biologics*								
**De Benedetti, 2021/2022, multicentre (Europe and USA)** **(5 reports)** [21, 37–40**]**	**Single-arm pilot study**	**Emapalumab started at 6 mg/kg, continued 3 mg/kg every 3 days until day 15, then twice weekly until day 28**	**i.v. corticosteroids (14), ciclosporin (8), anakinra (7), IVIG (3)**	**Ciclosporin in 6, anakinra in 5**	**14 patients aged 2–25 (median 11 years)** **1 adult-onset Still’s disease**	**Duration unclear; age at onset 1–17 years**	**All alive at 12 months**	**5× CMV reactivation/infection**
							**Remission of MAS (resolution of clinical signs/symptoms and normalization of lab parameters) in 13/14; recurrence of MAS in 1 patient at 12 months** **6 flares of Still’s within the first 8 weeks, 4 further flares over the next 10 months**	
							**Steroid dose reduced by >/=50% in 12/14 by 8 weeks**	
Chellapandian, 2023, USA (2 reports) [52, 53]	Case report	Emapalumab, started at 6 mg/kg (1 dose), continued at 3 mg/kg twice weekly for 4 weeks	i.v. and oral corticosteroids, anakinra, MTX, tocilizumab, and canakinumab	Corticosteroids	1 patient, aged 5 years	Duration 3 years; age at onset 21 months	MAS remission (resolution of symptoms with improved ferritin) Then went on to have allogenic HSCT after reduced-intensity conditioning. Complete resolution of Still’s at 20 months, with improved lung disease.	None reported
**Kostik, 2022, Russia** [44**]**	**Case series**	**Canakinumab increased dose of 150–300 mg (3–7.5 mg/kg) fortnightly, then monthly**	**Corticosteroids, IVIG, tocilizumab in 2, canakinumab in 2**	**Corticosteroids; tofacitinib in 1, ciclosporin in 1**	**4 patients, age at intervention unclear**	**Duration unclear; age at onset 1–9 years**	**All alive (follow-up duration unclear)**	**No observed side effects**
							**Clinical remission on treatment in 2/4, minimal disease activity in 1/4, resolution of recurrent MAS but development of lung disease in 1/4**	
Yasin, 2017, 2018, USA [45, 46]	Case report	IL-18 blockade with Tadekinig alfa	Not described	Not described	1 patient, aged 5 years	Not reported	Steroid dose reduced by >50%; milder episodes of recurrent MAS	Lung disease—unclear if drug-related
Bracaglia, 2023 [47]	Case report	MAS825, an IL-1 and IL-18 neutralizing antibody	Corticosteroids, anakinra, emapalumab	Prednisolone	1 patient, aged 9 years	Duration 6 years; age at onset 3 years	Clinical remission (rapid improvement of clinical and laboratory parameters) sustained for 10 months’ follow-up	None reported
							Off corticosteroids	
*Combination therapies*								
Slaney, 2024, USA [73]	Case report	Anakinra (high dose), emapalumab, etoposide	i.v. methylprednisolone, anakinra, tacrolimus, ciclosporin	i.v. corticosteroids	1 patient, aged 2 years	Duration 1 year; age at onset 13 months	Off corticosteroids at 24 months with no recurrence of MAS	None reported
*Haematopoietic stem cell transplant*								
**Silva, 2018, UK** [62**]**	**Multicentre case series/cohort study**	**Allogenic HSCT**	**Corticosteroids, ciclosporin in 4, MTX in 2, etoposide in 1, anakinra in 4, tocilizumab in 1, anti-thymocyte globulin in 1**	**Conditioning regime** **Ciclosporin** **MMF**	**5 patients, aged 2.5–13 years**	**Duration 1–5 years; age at onset 5 months–12 years**	**Survival in 4/5 at minimum 2 years f/u**	**1 death due to fungal sepsis, viral infection/reactivation in 4/5, GVHD in 3, severe bacterial infection in 1, atypical mycobacterial infection in 1, pancytopaenia in 1**
							**Complete remission (symptom-free and off immunosuppressants) in 4/5**	
Chandra, 2016, USA [57]	Case report	Allogenic HSCT	Corticosteroids, ‘conventional and novel treatments’	Conditioning + ciclosporin, corticosteroids	2 patients, aged 4 and 16 years	Not reported	Alive at 6 months and 1 year	None reported
							No recurrence of Still’s	
Bracaglia, 2023, Rome [58]	Case report	Allogenic HSCT	i.v. methylprednisolone, increased dose anakinra, ciclosporin, emapalumab	Conditioning Anakinra	1 patient, 17 years	Duration 4 years; age at onset 13 years	Complete remission on anakinra (clinical condition and normal IL-18 and CXCL9) at 30 months	None reported
Davidson, 2021, USA [59]	Case report	Allogenic HSCT	Adalimumab, tocilizumab, prednisolone, IVIG, anakinra, canakinumab, leflunomide, MTX, ciclosporin, tacrolimus	Reduced intensity conditioning, tacrolimus, rituximab, methylprednisolone	1 patient, aged 21 at the time of HSCT	Duration 5 years; age at onset 16 years	Alive at 3 years, in remission (full range of movement of joints and no symptoms of immunosuppressants)	GVHD, febrile neutropaenia, hyperglycaemia, EBV reactivation, temporary B cell aplasia
**Jaime-Perez, 2022, Mexico** [63**]**	**Case series**	**Autologous HSCT**	**Corticosteroids, MTX, tocilizumab, etoposide**	**Conditioning** **Ciclosporin**	**1 patient with Still’s-MAS, aged 2 years**	**Duration 0.75 years; age at onset 1 year**	**Alive at 12 months’ follow-up**	**Neutropaenic sepsis, mucositis**
							**Complete remission (resolution of symptoms off immunosuppressive therapy)**	
*Other approaches*								
Liquidano-Perez, 2023, Mexico [48]	Case report	Plasma exchange	Dexamethasone, IVIG, ciclosporin, etoposide, tocilizumab	Dexamethasone, IVIG, ciclosporin, etoposide	1 patient, aged 5 years	Duration 7 weeks; age at onset 5 years	‘Clinical improvement’, improved cytopaenias but persistently high ferritin and triglycerides	None
Kinjo, 2018, Japan [49]	Case report	Plasma exchange, leukocytapheresis, and plasma diafiltration	IVIG, methylprednisolone, dexamethasone, ciclosporin, plasma exchange alone, infliximab	Methylprednisolone, ciclosporin, tocilizumab after 15 days	1 patient, aged 1 year	Duration 1.5 months; age at onset 1 year	Plasma exchange alone ineffective; ‘fever resolved and serum levels of proinflammatory cytokines decreased’; 'remission sustained on (oral) steroid and ciclosporin’—unclear dose or duration	None 'severe’
Sato, 2022, Japan [50]	Case report	Plasma exchange	Corticosteroids, MTX, ciclosporin, tocilizumab, canakinumab, IVIG	Unclear	1 patient, aged 6 years	Duration 1 year; age at onset 5 years	Fever improved but pancytopaenia worsened, requiring GM-CSF Improved CT chest, has 'remained well’ for 5 years	None reported
							Prednisolone reduced to 9 mg/day	

Pilot study and case series shown in bold. GVHD: graft-versus-host-disease; HSCT: haematopoietic stem cell transplant; MAS: macrophage activation syndrome; sJIA: systemic juvenile idiopathic arthritis; VAS: visual analogue scale.

#### Non-biologic medications

One case series of patients with refractory MAS+/− lung disease treated with moderate dosing of the chemotherapy agent etoposide (a mainstay of treatment in primary haemophagocytic lymphohistiocytosis (HLH) [36]) included five patients with Still’s [37]. All patients were alive and off corticosteroids at 2 years. All had a ‘complete response’ initially, but 1/5 developed recurrent MAS, going on to have an allogenic HSCT. Adverse effects reported were neutropaenic fever/sepsis in 2/5.

Ciclosporin is routinely used in Still’s, particularly with MAS, as discussed above. A single report meeting the chosen criteria for refractory Still’s was found, reporting no benefit [38]. Full texts of several studies using ciclosporin, tacrolimus and thalidomide in resource-limited settings, indicating some efficacy, were reviewed, but these did not meet the present definition of refractory Still’s.

#### JAK inhibitors

One case report of difficult-to-treat Still’s-MAS reported an initial response to tofacitinib, before recurrence of Still’s rash [39].

#### Biologics

##### Emapalumab

Emapalumab, an anti-IFN-gamma antibody, has been approved for use in primary HLH; high circulating levels of this cytokine in Still’s-MAS provide a target for therapeutic blockade [25]. A single-arm, multicentre pilot study of emapalumab in Still’s-MAS not responding to high-dose i.v. glucocorticoids reported outcomes of survival, remission of MAS and steroid dose at 8 weeks and 12 months [25, 40–43]. One patient with AOSD was included. Half were already on ciclosporin and/or anakinra, with continuation permitted. All patients survived. Remission of MAS (by clinical and laboratory criteria) was initially achieved in 13/14; one patient had a recurrence. Steroid dose was reduced by at least 50% in 12/14 patients by 8 weeks, and by 12 months, 11 patients were on no or very low dose steroids. Overall, emapalumab appeared efficacious in treating MAS, but did not prevent relapses of Still’s, with six flares by 8 weeks and four further flares over the remaining follow-up. Five patients continued anakinra, none having early flares, suggesting combination therapy may be required. There were five cases of CMV reactivation or infection.

##### Increased dose of IL-1 antagonist

One case series of increased dosing of canakinumab described four patients with refractory Still’s-MAS [44]. All survived the duration of follow-up, with remission in two and minimal disease activity in 1, although one developed lung disease whilst on canakinumab treatment.

##### IL-18 blockade

One case report of IL-18 blockade (Tadekinig alfa) for recurrent MAS requiring long-term steroids described that the steroid dose could be reduced by >50% and episodes of MAS became milder [45, 46].

##### MAS825

MAS825 (IL-1/IL-18 blockade), a novel dual-action biologic, was trialled in two cases, one with refractory Still’s-MAS [47]. Clinical remission was sustained for follow-up of 10 months, with corticosteroids stopped. This could represent a promising strategy, with a clinical trial awaited.

#### Other approaches

Three case reports of plasma exchange to treat refractory Still’s-MAS, one with accompanying lung disease, had mixed results [48–50]. All survived, with some improvement in symptoms, but ongoing need for steroids.

### Still’s-associated lung disease

Table 5 summarizes articles reporting treatments for refractory Still’s-associated lung disease.

**Table 5.** Treatments for Still’s-associated lung disease

Still’s lung disease								
Study (author, year)	Type of study	Intervention	Previous treatment	Concomitant medications	Intervention (number of patients; age)	Duration of illness (range)/age at diagnosis (range)	Outcomes	Adverse effects
*Non-biologic medications*								
**Horne, 2021, Sweden [** 24 **]**	**Case series**	**Etoposide, moderately dosed (50–100 mg/m^2^ once weekly)**	**i.v. methylprednisolone, oral corticosteroids, anakinra (3), ciclosporin (2)**	**Oral corticosteroids, ciclosporin (3), IVIG (2), anakinra (1), rituximab (1)**	**3 patients; 5 months—16 years**	**Duration 3–7 months**	**All alive at 2 years**	**Neutropaenic sepsis in 1**
							**‘Complete response’ in all 1 developed recurrent MAS->Allogenic HSCT** **‘Full pulmonary recovery’**	
							**All off corticosteroids at last follow-up (2–8 years after onset)**	
*JAK inhibitors*								
Bader-Meunier, 2020, France [51]	Case report	Ruxolitinib 1 mg/kg/day	Corticosteroids, anakinra, canakinumab, tocilizumab	i.v. and oral corticosteroids	1 patient, aged 4 years	Duration 3 years; age at onset 1 year	At 15 months, no fevers, CRP normal Chest CT abnormalities decreased, SpO_2_ improved from 92% to 100%	None reported
							Prednisolone reduced to 0.1 mg/kg/day	
*Biologics*								
Chellapandian, 2023, USA (2 reports) [52, 53]	Case report	Emapalumab, started at 6 mg/kg (1 dose), continued at 3 mg/kg twice weekly for 4 weeks	IV and oral corticosteroids, anakinra, MTX, tocilizumab, and canakinumab	Corticosteroids	1 patient, aged 5 years	Duration 3 years; age at onset 21 months	MAS remission (resolution of symptoms with improved ferritin) Then went on to have allogenic HSCT after reduced-intensity conditioning. Complete resolution of Still’s at 20 months, with improved lung disease (significant improvement on CT chest, no pulmonary symptoms)	None reported
Rood, 2023, USA [54]	Case report	MAS825, IL-1 and IL-18 neutralizing antibody	Corticosteroids, canakinumab, anakinra, MTX, cyclophosphamide, azathioprine, tofacitinib	Tofacitinib, prednisolone	1 patient, aged 10 years	Duration 8 years; age at onset 22 months	Resolution of respiratory symptoms; post-exercise SpO_2_ increased from 91% to 97%	None reported
							Prednisolone stopped at 10 months	
*Other therapies*								
Sato, 2022, Japan [50]	Case report	Plasma exchange	Corticosteroids, MTX, ciclosporin, tocilizumab, canakinumab, IVIG	Unclear	1 patient, aged 6 years	Duration 1 year; age at onset 5 years	Fever improved but pancytopaenia worsened, requiring GM-CSF Improved CT chest, has 'remained well’ for 5 years	None reported
							Prednisolone reduced to 9 mg/day	

Case series shown in bold. HSCT: haematopoietic stem cell transplant; MAS: macrophage activation syndrome; sJIA: systemic juvenile idiopathic arthritis.

#### Non-biologic medications

The case series of patients with refractory MAS treated with moderate dosing of etoposide included three patients with concurrent Still’s lung disease. One went on to have an allogenic HSCT due to recurrent MAS. All were reported to be off steroids at 2–8 years’ follow-up, with ‘full pulmonary recovery’.

#### JAK inhibitors

One case report of a patient with Still’s-associated lung disease reported improvement of CT chest abnormalities and baseline oxygen saturations with ruxolitinib, with prednisolone reduced to a low dose [51].

#### Biologics

Chellapandian reports a case of recurrent MAS + lung disease in Still’s treated with emapalumab, resulting in remission of MAS [52, 53]. This patient then had an allogenic HSCT, with no active Still’s and improved lung disease at 20 months.

MAS825, an IL-1 and IL-18 neutralizing antibody, was trialled in a case of Still’s-associated lung disease [54]. Tofacitinib was continued. The patient had resolution of respiratory symptoms and increased post-exercise SpO_2_, with tofacitinib stopped at 2 months and prednisolone at 10 months.

#### Other approaches

A patient with Still’s-MAS and associated lung disease treated with plasma exchange had improvement in CT chest findings but remained on 9 mg/day of prednisolone at 5 years [50]. It was unclear in the report which other treatments were continued.

### Haematopoietic stem cell transplant and other cell-based therapies

Bone marrow transplantation has been used to treat refractory Still’s where other treatment options have been exhausted, both before and after the advent of biologics.

Several case reports described patients with refractory Still’s arthritis and MAS who had allogenic HSCT [55–61]. All survived, and only one patient, with refractory arthritis, relapsed, though reporting bias likely affects the cases published. Significant morbidity was reported, including graft-versus-host-disease (GVHD), renal failure and viral reactivations.

A multicentre case series of patients with JIA treated with allogenic HSCT included 11 patients with Still’s, either refractory arthritis or MAS [62]. 10/11 survived to at least 10 months’ follow-up, with one death due to fungal sepsis. Complete remission was achieved in 8/11 patients (no recurrence after stopping immunosuppression), with partial remission in 2/11. One patient with Still’s arthritis required ongoing corticosteroids, whilst another flared at 22 months, requiring a short course of corticosteroids. Regarding adverse effects, 6/11 had viral infection/reactivation, 4/11 suffered GVHD, one developed severe bacterial infection, and two had haematological toxicities.

Two cases of patients with refractory Still’s arthritis undergoing autologous HSCT were identified, one included in a wider case series [63, 64]. Both survived but remained on corticosteroids, one after a relapse. Another patient in the case series, with Still’s-MAS, achieved clinical remission off immunosuppressants at 12 months of follow-up [63].

A single case of refractory Still’s arthritis treated with mesenchymal stromal cell infusion did not find this to be effective in this patient [65].

## Discussion

As therapeutic options for paediatric Still’s have improved over recent decades, a subset of patients with refractory disease have emerged. This represents a challenging cohort of patients for whom new treatment strategies are acutely needed [66].

The evidence base for treatment of refractory Still’s is limited, with most studies found being low-moderate quality, level 4 evidence consisting of case reports and case series, with unclear documentation of whether these included all eligible patients [26, 67]. Case reports are by nature subject to reporting bias, with positive results more likely to be reported, but can provide proof-of-concept and indicate areas for future research. There was also heterogeneity in use of diagnostic criteria for Still’s and MAS, and in outcome measures reported. In addition, most patients with refractory disease have had recent or concurrent treatment with a variety of other agents, which could confound results. Small numbers and lack of control groups limit the use of statistics to represent any effect size.

Research into such a rare, life-threatening condition is challenging. Including a placebo arm or washout period may not be safe or ethically acceptable. A withdrawal design has been used in previous studies in Still’s, but patient groups have also expressed reservations about this, with concern about lasting effects of flares during the withdrawal period [66]. Recruitment is another challenge, with multicentre research required. The 4th NextGen Therapies for sJIA and MAS symposium (2022) discussed the challenges and possibilities around conducting trials in this patient group and advocated for the use of historic control cohort, although this may not be acceptable to regulatory bodies [24, 66].

Guidance on management of Still’s has been produced by the CARRA group, American College of Rheumatology and the German Pro-Kind initiative, focusing on treat-to-target approaches with proactive introduction of IL-1 or IL-6 blockers, but these only briefly address refractory disease [68–70]. A recent paper proposed an algorithm for the management of Still’s in the UK [27]. If IL-1 and IL-6 blockade fails, trial of ‘other biologics’ (including anti-TNFs, abatacept and JAK inhibitors), plus addition of ciclosporin, is suggested, with HSCT a last resort. No studies of anti-TNFs were included here as none met the chosen definition of refractory Still’s, most being before the advent of IL-1/IL-6 inhibitors.

The present review focused on patients diagnosed in childhood, but the recently published EULAR/PreS guideline on management of systemic JIA and AOSD, advocating for consideration of these conditions as one disease, provides opportunities for translating research from adult practice to paediatric disease, as well as enhancing recruitment to prospective studies by including paediatric and adult patients. The guideline also provides a simple definition for CID: absence of Still’s disease-related symptoms and normal ESR/CRP, with remission being a period of at least 6 months with CID. If adopted, this could standardize outcome reporting and enhance the ability to collate evidence from different sources via meta-analysis. The guideline recommends that difficult-to-treat patients should be discussed with centres with expertise.

For refractory Still’s arthritis, the evidence signals some efficacy of JAK inhibitors, rituximab and combination biologics, though unfortunately, there was not consistent reporting of outcomes, despite available criteria for remission of Still’s arthritis [71]. In the small number of patients represented, only three achieved complete remission, all with JAK inhibitors. This may remain an elusive goal in patients with longstanding disease, with many of these patients having a long disease course, potentially having missed the ‘window of opportunity’. Dual biologic therapy may be effective for some; this is represented by papers reporting small numbers treated with combinations of anakinra plus abatacept (*n* = 4), tocilizumab plus ixekinumab (*n* = 1), or tofacitinib and IL-1/IL-6 inhibitors (*n* = 3).

The only prospective trial found was in the Still’s-MAS group [25]. This had promising results for emapalumab in inducing MAS remission, but suggested less effect on underlying Still’s. There were also suggestions of positive results with etoposide and with MAS825, an IL-1/IL-18-neutralizing antibody.

Allogenic HSCT has been reported as having almost curative results in small numbers of patients with both refractory Still’s arthritis and Still’s-MAS, though with some relapses. This treatment has risk of significant morbidity and mortality, so it is likely to remain a last resort.

There was very little evidence found for the treatment of Still’s-related lung disease, though positive results were reported with etoposide and for single patients treated with ruxolitinib and with MAS825.

There are a few ongoing trials in this patient group. The EMERALD study (NCT05001737) is a phase 3 open-label trial testing emapalumab in refractory Still’s-MAS [72]. Two trials are registered of JAK inhibitors in Still’s arthritis: NCT04088396 with one arm comparing baricitinib to tocilizumab, and NCT03000439, a randomized withdrawal study of tofacitinib.

This review collates evidence on a recently defined challenging patient group. Currently, the available evidence is diverse, uncontrolled and largely observational, with small numbers of patients, so it was not possible to pool any data. The definition of refractory Still’s used is relatively new and has not been universally accepted. The question posed in this review concerns a small but varied and challenging to manage group of patients.

A variety of potential therapeutic options are now available for treatment of Still’s and its refractory subtypes, though the evidence base is currently limited. Innovative clinical trials are required to provide evidence in these rare subgroups. The ultimate goal of future research may be personalized precision medicine based on a combination of predictive features (clinical, demographic and genetic) and immunophenotyping so that the most effective treatment for individual patients may be established early in the disease course.

## Supplementary Material

rkaf123_Supplementary_Data

## Data Availability

No new data were generated or analysed in support of this research.
