# Supplementary material for: Pharmacological and non-pharmacological treatments for refractory paediatric Still’s disease: a scoping review
Source: Rheumatol Adv Pract. 2025 Nov 18;9(4):rkaf123. doi: 10.1093/rap/rkaf123 (PMC12631771; doi:10.1093/rap/rkaf123)
Supplement: rkaf123_Supplementary_Data [file rkaf123_supplementary_data.zip › Supplementary Data S1_Scoping_Review_Protocol.docx]

# Supplementary Data S1. Review Protocol

## Question

**Topic:** Pharmacological and non-pharmacological treatments for refractory systemic juvenile idiopathic arthritis (sJIA, or Still’s disease)

**Question:** What is the evidence-based management of refractory sJIA, defined as (1)

(1) Refractory sJIA arthritis: failure to respond to IL-1 and/or IL-6 blockade, requiring ongoing corticosteroids with persistence of arthritic or systemic features

OR

(2) sJIA-related MAS requiring long-term corticosteroids/ recurrent (>/= 2 episodes of) sJIA-related MAS

OR

(3) sJIA LD: development of sJIA-associated lung disease?

**P**: Children and young people with refractory sJIA

**I:** Treatments including additional conventional DMARDs, TNF-inhibitors, rituximab, abatacept, IL-18 inhibitors, JAK inhibitors and bone marrow transplant

**C:** Other treatments

**O:** Survival, disease activity/remission, adverse effects

*IL-1 and IL-6 inhibitors have transformed the management of systemic JIA, but a subset of patients still have refractory disease, with persistent arthritis, macrophage activation syndrome or interstitial lung disease(1).*

*We plan to perform a scoping review of evidence for management of this refractory disease. Inclusive selection criteria will be used as available evidence is likely to include small studies and case series, with the aim to summarise and analyse the current evidence for different treatment options.*

1. *Erkens R, Esteban Y, Towe C, Schulert G, Vastert S. Pathogenesis and Treatment of Refractory Disease Courses in Systemic Juvenile Idiopathic Arthritis: Refractory Arthritis, Recurrent Macrophage Activation Syndrome and Chronic Lung Disease. Rheum Dis Clin North Am. 2021 Nov;47(4):585-606. doi: 10.1016/j.rdc.2021.06.003. Epub 2021 Aug 21. PMID: 34635293.*

## Inclusion and Exclusion Criteria

**Inclusion Criteria**

Children and young people with refractory sJIA, as defined above

Pharmacological and non-pharmacological treatments for this condition

After advent of IL-1 & IL-6 blockade in sJIA (approx. 2005/2010)

Include uncontrolled trials, retrospective data and case series/reports (as likely to be few RCTs)

English language

**Exclusion Criteria**

Adult-onset Still’s disease; non-systemic JIA; other conditions

Referring to diagnosis/disease course/pathophysiology/basic science

Review papers; conference proceedings

Criteria for refractory not met

Non-English language

## Search Strategy

**Databases searched:**

Medline, Embase (using OVID platform)

Cochrane database

*Supplementary Table S3: Detailed search strategy for Medline.*

**Ovid MEDLINE(R) ALL <1946 to April 15, 2024>**

| 1 | Arthritis, Juvenile/ | 11971 |
| --- | --- | --- |
| 2 | (juvenile idiopathic arthritis or still's disease).ti,ab,kf. | 9860 |
| 3 | 1 or 2 | 16221 |
| 4 | ((difficult* or hard or fail* or no) adj3 (treat* or respon*)).ti,ab,kf. | 336345 |
| 5 | refractory.ti,ab,kf. | 163885 |
| 6 | 4 or 5 | 489894 |
| 7 | 3 and 6 | 828 |
| 8 | limit 7 to english language | 759 |
| 9 | systemic.ti,ab,kf. | 607174 |
| 10 | 8 and 9 | 349 |

<https://ovidsp.ovid.com/ovidweb.cgi?T=JS&NEWS=N&PAGE=main&SHAREDSEARCHID=3rg6QoLFGuHnFGChrz4c0wNUs50MxzEvLxNWM0WeBeRhDsD7cOBYH84W1cPTfOMjM>

*Supplementary Table S4: Detailed search strategy for Embase.*

**Embase <1974 to 2024 April 15>**

| 1 | systemic juvenile idiopathic arthritis/ | 2248 |
| --- | --- | --- |
| 2 | (systemic juvenile idiopathic arthritis or still's disease).ti,ab,kf. | 5265 |
| 3 | 1 or 2 | 6194 |
| 4 | ((difficult* or hard or fail* or no) adj3 (treat* or respon*)).ti,ab,kf. | 513184 |
| 5 | refractory.ti,ab,kf. | 278295 |
| 6 | 4 or 5 | 768852 |
| 7 | 3 and 6 | 704 |
| 8 | limit 7 to english language | 662 |
| 9 | adult.ti. | 258216 |
| 10 | 8 not 9 | 382 |

<https://ovidsp.ovid.com/ovidweb.cgi?T=JS&NEWS=N&PAGE=main&SHAREDSEARCHID=b9EPK6JJAY0zlKBjQEY2Tb4NuNVmfEKg5NCaomacUN1qK5JHZpglCQk47dOPCvJN>

**Cochrane Database**

(systemic juvenile idiopathic arthritis AND refractory):ti,ab,kw (Word variations have been searched)

*Acknowledgements to Seona Hamilton, specialist librarian, for assistance with this search strategy.*

## Study Screening and Selection

Endnote reference manager used to collate and screen references**.**

**Search history:**

Medline – 349

Embase – 382

Cochrane – 31

Total – 762

14 Duplicates removed by Endnote, leaving 748

122 Removed by Deduplicator, leaving 626

557 Excluded at title/abstract, leaving 69 for full text review

## Data Collection

Tabulate data

*Supplementary Table S5: Preliminary data collection proforma.*

| Study (Authors, year) | Type of study | Definition of Refractory | Intervention (Number of patients) | Comparison (Number of patients) | Outcomes (Clinically inactive disease/survival) | Adverse effects |
| --- | --- | --- | --- | --- | --- | --- |
|  |  |  |  |  |  |  |
|  |  |  |  |  |  |  |
|  |  |  |  |  |  |  |

Separate tables for each drug class/intervention type

## Headings for Review

Abstract

Background (300 words)

Objectives (50 words)

Methods

PICO; Inclusion & Exclusion Criteria (150 words)

Search Methods (200 words)

Selection of Studies (150 words)

Results

PRISMA Diagram (50 words)

Excluded studies (100 words)

Risk of Bias in Included Studies (150 words)

Non-biologics (100 words)

Rituximab (100 words)

Anti-TNF therapies (100 words)

Emapalumab (Anti-IFNϒ) (100 words)

JAK inhibitors (100 words)

Stem cell/Bone marrow transplant and other cell therapies (100 words)

Plasma exchange (100 words)

Combination treatments(100 words)

Increased dose of IL-1 inhibitors (100 words)

Anti-IL-18 Binding Protein (100 words)

Ongoing studies? (100 words)

Discussion (750 words)

Total: Approximately 3000 words
