# Supplementary material for: Pharmacological and non-pharmacological treatments for refractory paediatric Still’s disease: a scoping review
Source: Rheumatol Adv Pract. 2025 Nov 18;9(4):rkaf123. doi: 10.1093/rap/rkaf123 (PMC12631771; doi:10.1093/rap/rkaf123)
Supplement: rkaf123_Supplementary_Data [file rkaf123_supplementary_data.zip › Supplementary_Material_S1,_S2 (2).docx]

| **Inclusion Criteria** | **Exclusion criteria** |
| --- | --- |
| Children and young adults with refractory systemic JIA/Still’s disease diagnosed <16 years, as defined in the text (Erkens et al, 2021) | Adult-onset Still’s disease; non-systemic JIA; other conditions |
| Pharmacological and non-pharmacological treatments for this condition | Referring to diagnosis/disease course/pathophysiology |
| After availability of IL-1 & IL-6 blockade | Review papers; conference proceedings |
| Include controlled trials, uncontrolled trials, retrospective data and case series/reports | Non-English language |

Supplementary Table S1: Inclusion and Exclusion Criteria

*JIA: juvenile idiopathic arthritis*

Supplementary Figure S2: PRISMA Diagram of Search Process

*JIA: juvenile idiopathic arthritis*

Records identified from:

Medline (n=349)

Embase (n=382)

Cochrane Library (n=31)

Total (n=762)

Records removed *before screening*:

Duplicate records removed by Endnote (n = 14)

Duplicate records removed by Deduplicator (n=122)

Records screened

(n = 627 )

Records excluded

(n = 556)

Reports assessed for eligibility

(n = 69)

Reports excluded:

Conference abstract with inadequate information (n=12)

Data included in another report (n=10)

Not meeting definition (n =7)

Predating IL-1/IL-6 blockade (n=4)

Not systemic JIA (n =2)

Study protocol (n=2)

Report of disease course (n=1)

Review (n=1)

Studies included in review

(n = 30)

Reports of included studies

(n = 37)

**Identification**

**Screening**

**Included**
